# Antibacterial ADP-ribosyl cyclase toxins inhibit bacterial growth by rapidly depleting NAD(P)^+^

**DOI:** 10.1016/j.jbc.2025.110491

**Published:** 2025-07-16

**Authors:** Jake Colautti, Youngchang Kim, John C. Whitney

**Affiliations:** 1 Michael DeGroote Institute for Infectious Disease Research, McMaster University, Hamilton, Ontario, Canada; 2 Department of Biochemistry and Biomedical Sciences, McMaster University, Hamilton, Ontario, Canada; 3 Structural Biology Center, X-ray Science Division, Advanced Photon Source, Argonne National Laboratory, Lemont, Illinois, USA; 4 David Braley Center for Antibiotic Discovery, McMaster University, Hamilton, Ontario, Canada

**Keywords:** bacterial toxin, cyclic ADP-ribose, X-ray crystallography, enzyme kinetics, toxin secretion

## Abstract

In metazoans, enzymes belonging to the bifunctional ADP-ribosyl cyclase/cyclic ADP-ribose (cADPr) hydrolase family regulate diverse cellular processes by synthesizing and hydrolyzing the intracellular second messenger cADPr, derived from the electron carrier NAD^+^. However, bacterial enzymes belonging to this family have not been characterized. Here, we identify a bacterial ADP-ribosyl cyclase that is associated with the type VII secretion system and functions as an antibacterial toxin. This enzyme, which we name Tac1, inhibits bacterial growth by rapidly hydrolyzing NAD^+^ and NADP^+^. We determine the X-ray crystal structure of Tac1 to a resolution of 1.4 Å, which reveals that this protein adopts the core catalytic fold of metazoan ADP-ribosyl cyclase enzymes such as CD38. Using a combination of biochemical and mutagenesis approaches, we identify catalytic residues within the active site of Tac1, which are responsible for the formation of a cADPr catalytic intermediate and subsequent hydrolysis of this intermediate into linear ADP-ribose. A bioinformatic analysis reveals that Tac1 is the founding member of a widespread family of bacterial ADP-ribosyl cyclase enzymes, many of which are associated with interbacterial conflict systems. We also identify enzymes in this family that are not associated with biological conflict systems and demonstrate that they produce cADPr as their major product rather than linear ADP-ribose, suggesting that these enzymes serve a biological function distinct from interbacterial antagonism. Together, these findings demonstrate that ADP-ribosyl cyclase/cADPr hydrolase enzymes function as toxins in diverse bacterial conflict systems and suggest that cADPr may play a previously overlooked role in bacterial physiology.

Many species of bacteria harbor specialized protein secretion systems that deliver antibacterial toxins into competitor cells, thus mediating competition for space and resources (1, 2). These so-called polymorphic toxins typically contain an N-terminal trafficking domain that enables their recognition and export by an associated protein secretion system and a C-terminal toxin domain that harbors lethal activity (3). Individual toxin domain families are often associated with multiple distinct trafficking domains in different bacterial species, which reflects the horizontal transfer of these toxin domains across bacterial genomes (3). In addition, these toxins are frequently encoded adjacent to cognate immunity proteins that bind to and inhibit their activity, which protects the producing organism from self-killing or killing of genetically identical kin cells. Thus, polymorphic toxins arm bacteria with an arsenal of functionally diverse “weapons” to selectively inhibit the growth of competitor, non-kin cells and compete for limited resources in their ecologic niche (4, 5, 6, 7, 8).

Several families of polymorphic toxins have been identified that kill target cells by depleting essential metabolites such as ATP or the electron carriers NAD^+^ and NADP^+^ (9, 10, 11, 12). By depleting these metabolites, these toxins starve target cells of energy carriers and thus inhibit catabolic and anabolic processes that are required for growth and survival (9, 11, 12, 13). Interestingly, toxins that act in this manner typically display extremely high catalytic rates. For instance, the NAD(P)^+^ glycohydrolase Tse6 hydrolyzes ∼10,000 NAD(P)^+^ molecules per minute, which enables a single molecule of this enzyme to deplete all available intracellular NAD(P)^+^ within minutes (12, 14). Similarly, the toxin Tas1 converts ATP into the metabolically inert products adenosine tetraphosphate and pentaphosphate at a rate of approximately 180,000 molecules per minute, thus depleting the cell of energy required for survival (9). The ubiquity of toxins that act by these mechanisms across diverse bacterial species suggests that the rapid depletion of essential metabolites represents a highly effective mechanism to inhibit competitor cell growth.

In addition to their role as electron carriers in redox metabolism, the dinucleotides NAD^+^ and NADP^+^ have been found to serve as substrates for the production of several intracellular signaling molecules across all domains of life (15). In metazoans, enzymes belonging to the ADP-ribosyl cyclase/cyclic ADP-ribose (cADPr) hydrolase (ARC) family catalyze the formation of N^1^- cADPr and nicotinic acid adenine dinucleotide phosphate from NAD^+^ and NADP^+^, respectively (16, 17, 18). These molecules serve as secondary messengers that mobilize intracellular stores of calcium, thus regulating several calcium-dependent physiological processes (17). While some members of the ARC family predominantly display cyclase activity, other enzymes in this family display additional cADPr/nicotinic acid adenine dinucleotide phosphate hydrolase activity (19, 20). These bifunctional enzymes therefore release linear ADP-ribose or ADP-ribose 2′-phosphate as the final product of reactions that proceeds through a cyclic intermediate. Despite decades of research into the physiology and biochemistry of metazoan ARC enzymes, to our knowledge, no enzymes belonging to this family have been characterized in bacteria nor has any bacterial enzyme been found to produce N^1^-cADPr.

Here, we demonstrate that enzymes belonging to the ARC family are widespread in bacterial genomes, where they generally function as antibacterial toxins. We discover an ADP-ribosyl cyclase/cADPr hydrolase (ARC) encoded within a bacterial type VII secretion system in *Streptococcus parasanguinis*, which we term Tac1, and demonstrate that this enzyme rapidly hydrolyzes NAD^+^ and NADP^+^ into ADP-ribose and ADP-ribose 2′-phosphate, respectively. A 1.4 Å crystal structure of Tac1 reveals that this protein shares striking similarity with metazoan ARC enzymes. Mutagenesis analysis guided by this structure enables identification of residues responsible for the cyclization of NAD^+^ into a cADPr intermediate and subsequent hydrolysis of this intermediate into linear ADP-ribose. Lastly, we bioinformatically identify Tac1 homologs that are not encoded in canonical interbacterial antagonism gene clusters and demonstrate that one such homolog produces cADPr as its major product, suggesting that a subset of bacterial ARC proteins do not function as toxins. Together, our findings demonstrate that ARC enzymes are widespread in bacterial conflict systems and suggest that cADPr may play a previously overlooked role in bacterial physiology.

## Results

### An antibacterial ARC enzyme associated with the type VIIb secretion system

**Figure 1:**
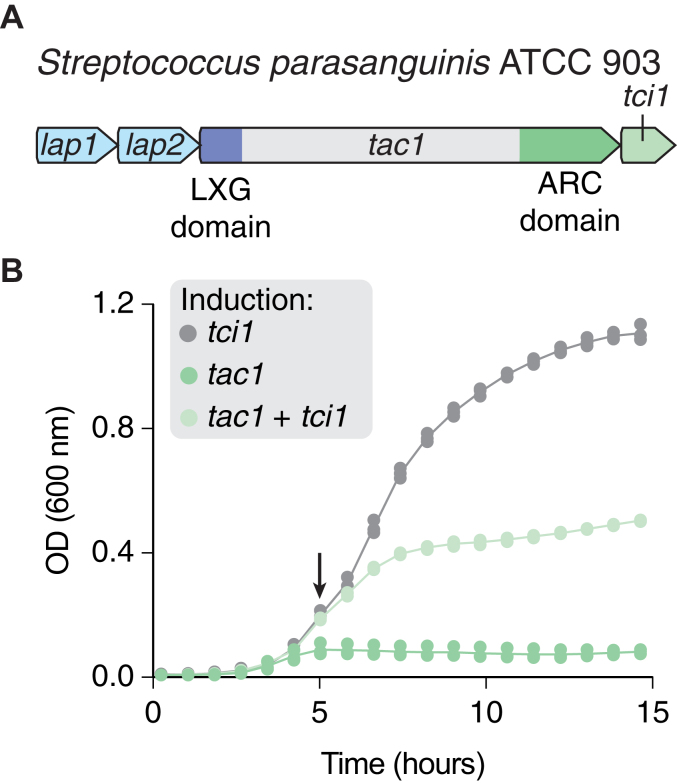
**Tac1 and Tci1 are an antibacterial toxin**-**immunity pair**. *A*, schematic representation of the Tac1-encoding gene cluster in *Streptococcus parasanguinis* strain ATCC 903. *B*, growth of *Escherichia coli* harboring plasmids encoding *tac1* and *tci1* under the control of distinct promoters. The *arrow* denotes the time at which the inducer for each gene was added. n = 3 biological replicates. ATCC, American Type Culture Collection.

In the course of studying the antibacterial type VIIb secretion system (T7SSb) in *Streptococcus* species, we identified an uncharacterized protein encoded by *S*. *parasanguinis* that contains a C-terminal ARC domain and an N-terminal Leu-X-Gly (LXG) domain (Fig. 1*A*) (21, 22, 23). LXG domains are a hallmark of antibacterial toxins secreted by the T7SSb, which suggests that this protein may too function as an antibacterial toxin (22, 23). While LXG domains are responsible for toxin export by the T7SSb, the antibacterial activity of these proteins is contained within enzymatic toxin domains, which are typically found at their C terminus (3, 23, 24). Therefore, the finding that an ARC domain is present at the C terminus of an LXG domain–containing protein suggests that this ARC domain likely functions as a bacterial toxin. T7SSb toxins are often encoded downstream of LXG-associated proteins (Lap), which are required for their recognition and export by the T7SSb secretion apparatus (21, 22). Consistent with this paradigm, this *S*. *parasanguinis* LXG domain–containing protein is encoded downstream of two Lap proteins, which further suggests that it functions as an antibacterial substrate of the T7SSb (Fig. 1*A*). Lastly, antibacterial toxins are invariably encoded adjacent to cognate immunity proteins that protect toxin-producing cells against their lethal activity (3, 23, 24, 25, 26, 27). Indeed, this LXG domain–containing protein is encoded adjacent to a protein belonging to the Imm74 superfamily, which has previously been hypothesized to function as a family of immunity proteins that inactivate antibacterial toxins (3). We therefore hypothesize that the *S*. *parasanguinis* ARC domain–containing protein is an antibacterial toxin and that its downstream open reading frame encodes a cognate immunity protein that neutralizes its toxic activity.

To test the hypothesis that these genes constitute an antibacterial toxin/immunity pair, we coexpressed the C-terminal ARC domain of this LXG protein and its adjacently encoded Imm74 protein under the control of distinct inducible promoters in *Escherichia coli*. Consistent with our prediction that this ARC domain acts as a toxin, isolated expression of this domain inhibits *E*. *coli* growth (Fig. 1*B*). Moreover, coexpression of the adjacently encoded Imm74 protein partially restores this growth defect (Fig. 1*B*). This finding, together with our observation that this ARC domain–containing protein is encoded within a canonical T7SSb toxin gene cluster, indicates that the ARC domain likely acts as a bacterial toxin. To reflect this activity, we renamed these genes toxic ADP-ribosyl cyclase 1 (*tac1*) and toxic ADP-ribosyl cyclase immunity 1 (*tci1*), respectively.

### ADP-ribosyl cyclase toxins rapidly hydrolyze NAD^+^ and NADP^+^

**Figure 2:**
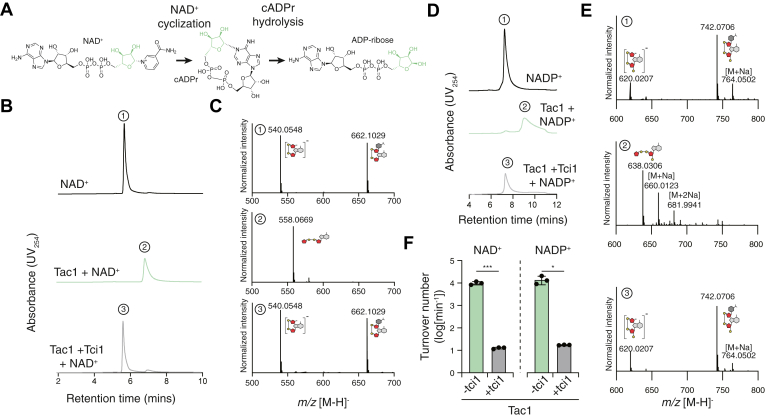
**Tac1 displays rapid NAD(P)^+^ hydrolase activity that is inhibited by Tci1**. *A*, schematic representation of the reaction catalyzed by bifunctional ADP-ribosyl cyclase/cADPr hydrolase enzymes. *B*, HPLC chromatograms of reaction products following incubation of NAD^+^ with either purified Tac1 alone or Tac1 with a molar excess of purified Tci1. *C*, mass spectra of the numbered HPLC peaks from (*B*). The mass of major species is indicated above the corresponding peak, and the species is identified by the *cartoon representation*. *D*, HPLC chromatograms of reaction products following incubation of NADP^+^ with either purified Tac1 alone or Tac1 with a molar excess of purified Tci1. *D*, mass spectra of the numbered peaks from (*D*). The mass of major species is indicated above the corresponding peak, and the species is identified by the *cartoon representation*. *F*, turnover of NAD^+^ or NADP^+^ by Tac1 in the presence or the absence of a molar excess of Tci1. Error bars represent mean ± SEM, n = 3 biological replicates. Mean differences were evaluated using an unpaired homoscedastic *t* test. The exact *p* value for the mean difference between Tac1 and Tac1–Tci1 is 0.0005 for NAD^+^ and 0.0140 for NADP^+^. Data in *B*–*F* are representative of at least three biological replicates.

Having established the antibacterial activity of Tac1, we next sought to determine the mechanism by which this protein inhibits bacterial growth. The ARC family is a multifunctional enzyme family capable of cyclizing NAD^+^ and NADP^+^ into cADPr and cADPr 2′-phosphate, respectively (Fig. 2*A*) (16, 18, 28). Some ARC family proteins are additionally capable of hydrolyzing these cyclic nucleotides into the linear products ADP-ribose and ADP-ribose 2′-phosphate (19, 20). Given the established role of cADPr in intracellular signaling in metazoans, we hypothesized that Tac1 produces cyclic nucleotides, which may inhibit bacterial growth by perturbing an as-yet unidentified signaling pathway as has been shown for other interbacterial toxins that produce nucleotide-derived signaling molecules (9). We also considered the alternative possibility that Tac1 does not release a cyclic product but instead rapidly hydrolyzes NAD(P)^+^, thus disrupting central metabolism, as is the case for antibacterial toxins belonging to the NAD(P)^+^ glycohydrolase family (11, 12). To discern between these possibilities, we first aimed to determine whether Tac1 displays predominantly cyclase or hydrolase activity. We therefore incubated purified Tac1 with NAD^+^ or NADP^+^ and analyzed reaction products by HPLC coupled to mass spectrometry (HPLC–MS). Incubation of Tac1 with NAD^+^ results in the formation of a major species at *m/z* 558.0669, consistent with the mass of linear ADP-ribose (Fig. 2*B*, *C*), whereas incubation with NADP^+^ results in the formation of a major species at *m/z* 638.0306, consistent with the mass of linear ADP-ribose 2′-phosphate (Fig. 2*D*, *E*). Both linear species are undetectable in reactions where an excess of purified Tci1 was included, confirming that this enzymatic activity is specific to Tac1 and that Tci1 inhibits Tac1 activity. This finding suggests that Tac1 functions predominantly as a bifunctional ARC and that the antibacterial activity of Tac1 is a consequence of NAD(P)^+^ depletion.

Previously characterized antibacterial NAD(P)^+^ glycohydrolase toxins display extremely rapid catalytic rates (10^4^–10^5^ min^-1^) and are thus capable of depleting all available cellular NAD(P)^+^ within minutes (11, 12). If Tac1 toxicity indeed results from NAD(P)^+^ depletion, then we might expect this enzyme to display turnover numbers comparable to that of toxic NAD(P)^+^ glycohydrolase enzymes. Indeed, a single molecule of Tac1 is capable of hydrolyzing approximately 12,000 NAD(P)^+^ molecules per minute (Fig. 2*F*). This rapid consumption of NAD(P)^+^ and the release of linear ADP-ribose and ADP-ribose phosphate species suggests that, like toxins belonging to the NAD(P)^+^ glycohydrolase family, Tac1 inhibits bacterial growth by depleting available NAD^+^ and NADP^+^.

### The X-ray crystal structure of a bacterial ADP-ribosyl cyclase enzyme

**Figure 3:**
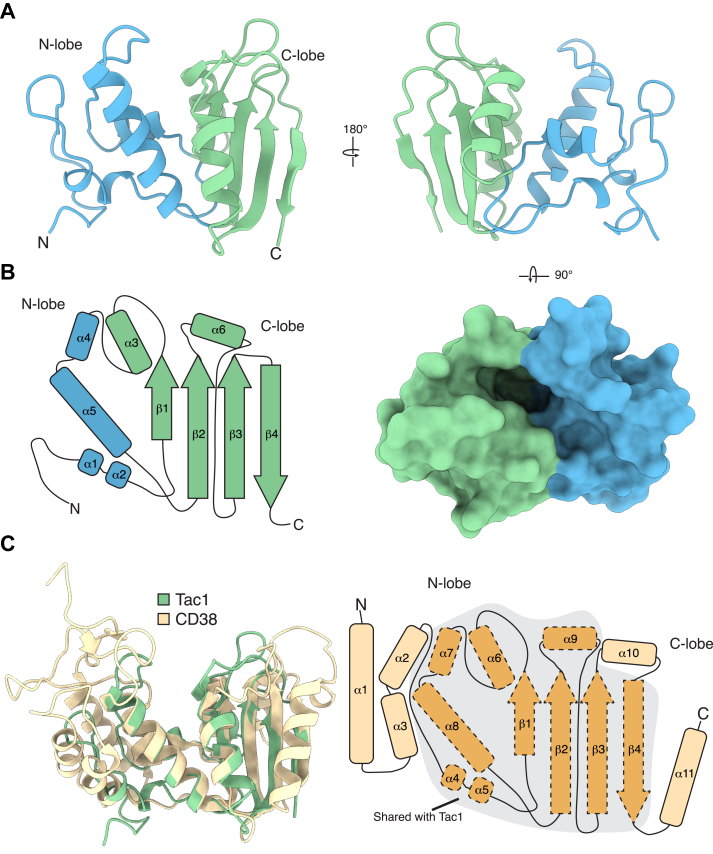
**X-ray crystal structure ofthe toxin domain ofTac1**. *A*, the overall structure of Tac1, with the N-terminal (*blue*) and C-terminal (*green*) lobes indicated. *B*, schematic representation of the secondary structure of Tac1. α-helices are denoted by *tubes*, and β-sheets are represented by *arrows*. The N- and C-terminal lobes are colored *blue* and *green*, respectively, according to the boundaries presented in (*A*). *C*, overlay of Tac1 (*green*) with human CD38 (*beige*; Protein Data Bank ID: 1YH3). A schematic representation of the secondary structure of CD38 is presented at *right*, with the secondary structures shared between CD38 and Tac1 outlined by *dashed lines*.

To better understand the molecular basis for Tac1 NAD(P)^+^ hydrolysis, we determined the X-ray crystal structure of this protein to a resolution of 1.4 Å (Fig. 3*A*, Table 1). Like metazoan ARC enzymes, Tac1 adopts a mixed α/β fold containing two lobes separated by a deep cleft (Fig. 3*A*, *B*). The N-terminal lobe of Tac1 is composed of four α-helices, whereas the C-terminal lobe is formed by a four-stranded antiparallel β-sheet flanked by two additional α-helices (Fig. 3*B*). This topology contrasts that of characterized metazoan ADP-ribosyl cyclase enzymes, which contain seven α-helices in their N-terminal lobe and three α-helices and a four-stranded antiparallel β-sheet in their C-terminal lobe (29, 30). This observation suggests that Tac1 may adopt the core catalytic fold of metazoan ARC enzymes but lacks additional secondary structure elements present in these enzymes.

**Table 1:** X-ray data collection and refinement statistics

	Tac1 (458-602)
Data collection	
Space group	P2_1_2_1_2
Cell dimensions	
*a*, *b*, *c* (Å)	57.19, 59.15, 40.08
*α*, *β*, *γ* (°)	90, 90, 90
Wavelength (Å)	0.97934
Resolution (Å)	33.18–1.39 (1.46–1.39)^a^
No. of reflection	28,179 (3879)
*R*_merge_^b^	0.117 (1.271)
*I*/σ(*I*)	9.7 (1.6)
Completeness (%)	99.4 (95.8)
Redundancy	11.2 (8.6)
CC_1/2_	0.998 (0.576)
Refinement	
Resolution (Å)	33.18–1.39 (1.43–1.39)
No. of reflections used	28,109 (2494)
Free (%)	4.9
*R*_work_/*R*_free_^c^	0.170/0.197
No. of protein chains/asymmetric unit	1
No. of atoms	1209
Protein	1198
Ligand/ion	11
Water	96
Wilson *B*-factor (Å^2^)	14.3
*B*-factors (Å^2^)	24.5
Protein	23.8
Ligand/ion	41.9
Water	31.3
RMSD	
Bond lengths (Å)	0.009
Bond angles (°)	0.943
MolProbity statistics^d^	
Clashscore	3.33
Rotamer outlier	1.50
Ramachandran plot	
Favored/outlier (%)	95.49/1.50
Protein Data Bank ID	9OUF

a Values in parentheses correspond to the highest resolution shell.

b *R*_merge_ = Σ Σ |*I*(*k*) - <*I*>|/Σ *I*(*k*), where *I*(*k*) and <*I*> represent the diffraction intensity values of the individual measurements and the corresponding mean values. The summation is over all unique measurements.

c *R*_work_ = Σ ||*F*_obs_| - *k*|*F*_calc_||/|*F*_obs_|, where *F*_obs_ and *F*_calc_ are the observed and calculated structure factors, respectively. *R*_free_ is the sum extended over a subset of reflections excluded from all stages of the refinement.

d As calculated using MolProbity (62).

To further explore the structural relationship between Tac1 and metazoan ARC proteins, we aligned our structure with an X-ray crystal structure of the enzymatic domain of the human bifunctional ARC CD38 (30) (Fig. 3*C*). Despite low pairwise sequence identity between these proteins (18%), our crystal structure strongly aligns with the catalytic cleft of CD38 (Cα RMSD of 0.97 Å across 67 equivalent positions) (Fig. 3*C*). This observation confirms our hypothesis that Tac1 adopts the core catalytic fold of CD38 and suggests that it may catalyze NAD(P)^+^ hydrolysis by a similar mechanism.

### NAD(P)^+^ hydrolysis proceeds through a cyclic intermediate

**Figure 4:**
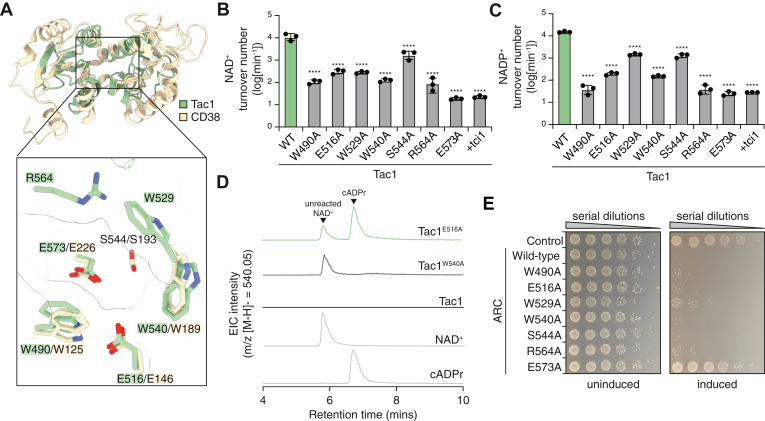
**The active site of Tac1 resembles that of metazoan ARC enzymes and Tac1 NAD(P)^+^ hydrolysis occurs through a cADPr intermediate**. *A*, overlay of Tac1 (*green*) with human CD38 (*beige*; Protein Data Bank ID: 1YH3), with active site residues highlighted in the *inset*. *B* and *C*, turnover of NAD^+^ or NADP^+^ by Tac1, the indicated Tac1 variants, or a control reaction containing an excess of Tci1. Error bars represent mean ± SEM, n = 3 biological replicates. Mean differences were evaluated using a one-way ANOVA with multiple comparisons to the wildtype enzyme. The adjusted *p* value for each comparison is <0.0001. *D*, HPLC–MS extracted ion chromatograms (*m/z* = 540.05) of reaction products following incubation of NAD^+^ with the indicated Tac1 variants. NAD^+^ and cADPr denote standards for HPLC retention times of these species (normalized to the intensity of cADPr in the Tac1^E516A^ reaction). *E*, growth of *Escherichia coli* harboring plasmids encoding the indicated *tac1* variants in the presence or the absence of inducer. Data in *B*–*E* are representative of at least two biological replicates. ARC, ADP-ribosyl cyclase/cyclic ADP-ribose hydrolase; cADPr, cADPr.

With an X-ray crystal structure of Tac1 in hand, we next set out to identify residues responsible for its cyclase and/or hydrolase activity. Metazoan ARC enzymes bind and act upon their substrates using the cleft between their two lobes (30). The high structural similarity between our bacterial ARC enzyme and CD38 led us to hypothesize that Tac1 uses a similar active site to metazoan ARC enzymes. Encouragingly, AlphaFold3 confidently predicts that the Tci1 immunity protein binds within this cleft in Tac1, supporting a role for this surface of the protein in NAD(P)^+^ hydrolysis (Fig. S1). Furthermore, several catalytic residues present in the active site of CD38 are conserved in Tac1, further suggesting a role for this region of the protein in NAD(P)^+^ hydrolysis (Fig. 4*A*). We therefore substituted the residues in this cleft that are conserved with CD38 with alanine and tested the capacity of these variants to hydrolyze NAD(P)^+^ (Fig. 4*B*, C). We additionally substituted the three tryptophan residues within this putative active site with alanine, since these residues are known to contribute to NAD(P)^+^ binding by interaction with the purine ring of these nucleotide substrates in CD38 (Fig. 4*A*) (31). Consistent with our hypothesis that this cleft forms the Tac1 active site, substituting any residue in this cleft with alanine profoundly reduced NAD(P)^+^ hydrolysis (Fig. 4*B*, C). However, only substitution of Glu573 with alanine completely abrogated NAD(P)^+^ hydrolase activity (Fig. 4*B*, C). Glu573 in our structure corresponds to Glu226 in CD38, which was previously found to be essential for the activity of this human enzyme (31). The striking similarity of the Tac1 active site with that of CD38 together with our finding that Tac1 Glu573 plays an essential role in catalysis confirms our hypothesis that Tac1 shares a conserved active site with metazoan ARC enzymes and therefore likely acts by a comparable mechanism.

Having identified several similarities between the active site of Tac1 and that of CD38, we next aimed to determine whether dinucleotide hydrolysis by Tac1 similarly proceeds through a cADPr intermediate. The cADPr hydrolase activity of bifunctional metazoan ARC enzymes requires a second glutamate residue on the opposite side of the active site relative to the essential catalytic glutamate (32). Substitution of this second glutamate (Glu146 in human CD38) with alanine favors the release of the cADPr catalytic intermediate over the complete hydrolysis of NAD^+^ into linear ADP-ribose (33). Encouragingly, Tac1 contains a second glutamate in its active site (Glu516) at a position corresponding to that of Glu146 in CD38 (Fig. 4*A*). This observation led us to hypothesize that Glu516 in Tac1 may play an analogous role in cADPr hydrolysis and that substitution of this residue with alanine may favor the release of the cADPr intermediate. We therefore analyzed the products of catalysis by Tac1^E516A^ using HPLC–MS and compared these products to those released by wildtype Tac1. HPLC–MS analysis of the products is complicated by the fact that cADPr has the same mass–charge ratio as an ionization fragment of NAD^+^ generated during MS. However, the different HPLC retention times for the two species allows for the differentiation of cADPr from NAD^+^ (Figs. 4*D*, S2*A*). As expected, wildtype Tac1 did not produce detectable cADPr, consistent with the complete hydrolysis of NAD^+^ into linear ADP-ribose by this enzyme (Figs. 4*D*, S2). However, incubation of NAD^+^ with Tac1^E516A^ led to the formation of a species with the same mass and HPLC retention time as a cADPr standard (Fig. 4*E*). Importantly, this species is not detected in a reaction catalyzed by Tac1^W540A^, which displays similar NAD(P)^+^ hydrolysis kinetics as Tac1^E516A^ (Fig. 4*D*). This finding confirms that the release of cADPr results from the specific substitution of Glu516 and is not simply a consequence of decreased NAD(P)^+^ hydrolase activity. We therefore conclude that like metazoan ARC enzymes, Tac1 hydrolyzes NAD^+^ through a cADPr catalytic intermediate in a manner that is enhanced by Glu516.

Last, we aimed to determine the consequences of these active site substitutions on the antibacterial activity of Tac1. To this end, we expressed these Tac1 variants in our heterologous *E*. *coli* expression system. Interestingly, despite catalytic activities that are orders of magnitude diminished relative to wildtype Tac1, most Tac1 variants retained their toxicity in this system (Fig. 4*E*). However, consistent with its completely abrogated NAD(P)^+^ hydrolase activity *in vitro*, expression of Tac1^E573A^ does not inhibit *E*. *coli* growth (Fig. 4*E*). The finding that even low levels of NAD(P)^+^ hydrolase activity inhibit bacterial growth likely reflects the essential nature of these dinucleotides, since even small perturbations to the cellular pool of NAD(P)^+^ may be incompatible with survival. Indeed, prior work on NAD(P)^+^ glycohydrolase toxins has shown that even variants with strongly diminished hydrolase activity *in vitro* retain toxicity when expressed in *E*. *coli* (11). Together, these results confirm our hypothesis that like its metazoan counterparts, the active site of Tac1 is formed by the cleft between its N- and C-terminal lobes and that this enzyme hydrolyzes NAD^+^ through a cADPr intermediate.

### ADP-ribosyl cyclase toxins are widespread among interbacterial conflict systems

Our results thus far suggest that bacterial ARC enzymes act as toxins that rapidly deplete cellular NAD(P)^+^. Since polymorphic C-terminal toxin domains are often found across multiple distinct toxin systems, these findings prompted us to examine the distribution of Tac1 homologs and explore their genetic contexts. To this end, we examined the gene neighborhoods of all ARC-annotated bacterial proteins in the RefSeq database (34). In addition, to minimize the possibility that Tac1-like enzymes are omitted from our analysis because of poor annotation, we additionally queried the toxin domain of Tac1 against the Genome Taxonomy Database and examined the gene neighborhoods of these Tac1 homologs (35).

**Figure 5:**
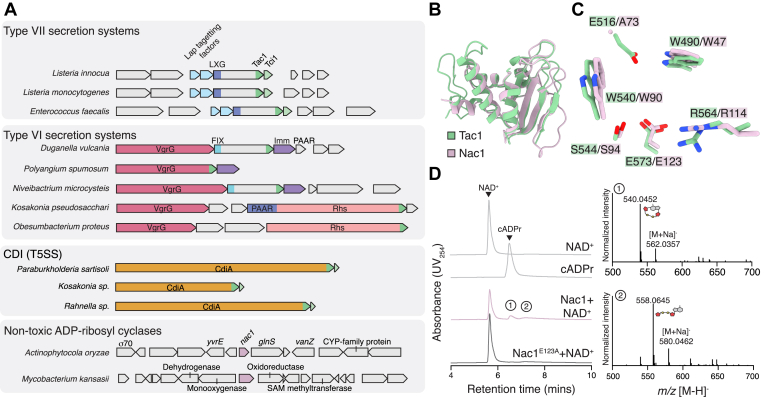
**Bioinformatic identification of toxic and nontoxic bacterial ARC enzymes**. *A*, schematic representation of gene neighborhoods encoding ARC enzymes in bacteria. Domains associated with each polymorphic toxin system are indicated: PAAR (proline-alanine-alanine-arginine), VgrG (valine glycine repeat G), FIX (phenylalanine-containing marker for type VI), Rhs (recombination hotspot), Lap (LXG-associated protein), and LXG (leucine-X-glycine). *B*, overlay of the Tac1 X-ray crystal structure (*green*) with an AlphaFold3-predicted model of *Actinophytocola oryzae* Nac1 (*purple*). *C*, comparison of the active site residues present in the crystal structure of Tac1 (*green*) with those present in the AlphaFold3 model of Nac1 (*purple*). *D*, HPLC chromatograms of reaction products following incubation of NAD^+^ with Nac1 or the catalytically inactive variant Nac1^E123A^. NAD^+^ and cADPr HPLC standards are included for the HPLC retention times of these species. Mass spectra of the numbered peaks are presented at the *right*. ARC, ADP-ribosyl cyclase/cyclic ADP-ribose hydrolase.

This gene co-occurrence analysis revealed that genes encoding ARC enzymes are present in a wide range of polymorphic toxin systems across diverse Gram-negative and Gram-positive bacteria (Fig. 5*A*). Perhaps unsurprisingly, we identified gene clusters in *Enterococcus* and *Listeria* species, which closely resemble the *S*. *parasanguinis* Tac1 gene cluster. This gene synteny is defined by the presence of two Lap-targeting factors, an LXG domain–containing protein with a C-terminal ARC domain and an immunity protein belonging to the Imm74 superfamily, a genetic architecture that is indicative of export by the T7SSb (Fig. 5*A*). In addition, we identified ARC domains in proteins associated with the antibacterial type VI secretion system (T6SS), a polymorphic toxin system widespread in Gram-negative species (36). Interestingly, these ARC domains are present in multiple distinct T6SS-associated proteins; in some instances, ARC domains are present at the C terminus of the secreted spike proteins VgrG and PAAR, whereas other ARC domains are present as distinct effector proteins that likely rely on adjacently encoded VgrG proteins for their T6SS-dependent export (Fig. 5*A*). Surprisingly, the gene downstream of these ARC proteins does not belong to the Imm74 family, suggesting that these T6SS-associated ARC toxins may be neutralized by an alternative immunity protein (Fig. 5*A*). Consistent with this possibility, AlphaFold3 confidently predicts that these downstream immunity proteins bind within the active site cleft of their associated ARC toxins, despite adopting a fold that shares no similarity with that of Tci1 (Fig. S3). Lastly, we identified several ARC domain–containing proteins belonging to the CdiA family of antibacterial toxins, a diverse family of polymorphic toxins that mediate contact-dependent inhibition between closely related Gram-negative bacteria (Fig. 5*A*) (1, 37, 38). Like those associated with the T7SSb, these CdiA-associated ARC toxins are encoded adjacent to Imm74-family immunity proteins. Together, these findings reveal that homologs of Tac1 are present in a range of functionally diverse polymorphic toxin systems harbored by bacteria belonging to multiple phyla, which suggests that the mechanism of NAD(P)^+^ depletion by Tac1 is likely widespread in interbacterial competition.

In addition to these putatively toxic ARC enzymes, we also identified Tac1 homologs that are not encoded alongside components of known polymorphic toxin systems (Fig. 5*A*). Although examination of the gene neighborhoods surrounding these homologs does not offer clues to their biological function, the absence of apparent immunity proteins encoded alongside these enzymes suggested to us that they may not act as antibacterial toxins. To reflect their potential alternative function, we tentatively named these enzymes nontoxic ADP-ribosyl cyclases (Nac). Interestingly, one such enzyme encoded by the rice commensal organism *Actinophytocola oryzae* is predicted to contain an N-terminal signal peptide that would target it for cotranslational export through the Sec translocon, indicating that it may function extracellularly (Fig. S4) (39). AlphaFold3 modeling of this enzyme, which we term Nac1, reveals that it is predicted to adopt a fold almost identical to that of Tac1 (Fig. 5*B*) (40). However, upon examining its predicted active site, we noticed that the residue corresponding to Glu516 in Tac1 is substituted with an alanine in Nac1 (Fig. 5*C*). Given the importance of Glu516 for cADPr hydrolysis by Tac1 (Fig. 4*D*), the absence of a glutamate in this position in Nac1 led us to hypothesize that Nac1 may display substantial ADP-ribosyl cyclase rather than predominantly NAD^+^ hydrolase activity. Because no bacterial enzyme has been found to natively produce cADPr, we sought to explore the biochemical possibility that Nac1 may synthesize this molecule. We therefore incubated NAD^+^ with purified Nac1 and analyzed the reaction products by HPLC–MS. Our ability to purify this enzyme from the cytoplasm of *E*. *coli* in the absence of any immunity protein supports our informatic hypothesis that it is not an antibacterial toxin. Furthermore, incubation of NAD^+^ with Nac1 led to the formation of both cADPr and linear ADP-ribose in approximately equal quantities (Fig. 5*D*). This activity contrasts with that of Tac1, which produces undetectable quantities of cADPr under the conditions of our assay. In addition, both the cyclase and hydrolase activities of Nac1 were abrogated when we substituted the catalytic residue Glu123 with alanine, confirming that these enzymatic activities are both specific to Nac1 (Fig. 5*D*). We therefore conclude that Nac1 produces significant levels of cADPr, which suggests that the enzyme’s biological function is distinct from that of toxic ARC enzymes and potentially implicates cADPr in bacterial physiology.

## Discussion

Here, we demonstrate that bacterial enzymes belonging to the ARC family function as antibacterial toxins that rapidly degrade the nucleotides NAD^+^ and NADP^+^. An X-ray crystal structure of one such enzyme, Tac1, reveals that these proteins share strong structural similarity with the core catalytic fold of metazoan ARC enzymes. Using a combination of biochemical and mutagenesis analyses, we identify the residues in Tac1 that are responsible for cyclization of NAD^+^ into a cADPr catalytic intermediate and subsequent hydrolysis of this intermediate into linear ADP-ribose. Lastly, we discover a subset of bacterial ARC enzymes that do not function as toxins and instead produce significant quantities of both cADPr and ADP-ribose. Together, our findings reveal a previously unappreciated role for ARC enzymes in bacteria and potentially implicate cADPr in the physiology of these organisms.

The importance of NAD(P)^+^ to all forms of cellular life, together with the intrinsic lability of these molecules, makes them an ideal target for cellular toxins. Indeed, bacterial toxins that target these molecules have been implicated both in competition between bacteria and in virulence toward eukaryotic hosts (11, 12, 41). However, only bacterial toxins belonging to the NAD(P)^+^ glycohydrolase family have been shown to display this catalytic activity (11, 12). We find that Tac1 adopts a different fold from these glycohydrolase enzymes and that NAD(P)^+^ hydrolysis by Tac1 proceeds through a cyclic intermediate. Therefore, ARC toxins and NAD(P)^+^ glycohydrolases act by distinct catalytic mechanisms but ultimately achieve the same cellular effect: inhibition of growth by depletion of the essential nucleotides NAD(P)^+^. Our finding that ARC enzymes are encoded in diverse polymorphic toxin systems suggests that this mechanism of NAD(P)^+^ depletion represents a widespread strategy of interbacterial competition. However, future studies will be needed to conclusively demonstrate that these Tac1 homologs function analogously to Tac1.

The striking structural similarity between Tac1 and human CD38, together with our finding that ARC enzymes are encoded by bacteria spanning multiple phyla, raises important questions about the evolutionary history of ARC proteins. Our finding that only a minority of bacterial genomes encode an ARC enzyme suggests that these enzymes were not vertically inherited from the common ancestor of both bacteria and eukaryotes. It therefore seems likely that the similarities between Tac1 and CD38 are a consequence of convergent evolution. This idea is supported by the widespread nature of ARC enzymes in polymorphic toxin systems, since toxin domains used by these systems are often acquired from unrelated organisms by horizontal gene transfer and subsequently incorporated into a species’ native toxin repertoire (3). Thus, evolutionary convergence of the Tac1 ancestor on the functional fold present in metazoan ARC enzymes likely did not occur in the common ancestor of all bacteria harboring a Tac1-like toxin. Instead, the widespread Tac1 homologs identified in our analysis likely arose from a limited number of evolutionary events and were distributed across bacterial taxa by horizontal gene transfer.

Our identification of nontoxic Tac1 homologs that display substantial ADP-ribosyl cyclase activity suggests that cADPr may play a previously overlooked role in bacterial physiology. Although the precise role of this molecule remains unknown, it is likely that cADPr serves as a signaling molecule in some bacterial species in a manner analogous to the role it plays in intracellular signaling in metazoans. In line with this possibility, several NAD^+^-derived cyclic nucleotides have recently been found to function as signaling molecules that coordinate bacterial defense against viral infection (42, 43, 44). In addition, cyclic nucleotides are known to govern a vast range of physiological processes in all domains of life, which further suggests that cADPr may serve a signaling function in bacteria (45, 46). Furthermore, our finding that Nac1 in *A*. *oryzae* contains an N-terminal signal peptide suggests that in some instances cADPr may function extracellularly. Secreted ARC enzymes such as Nac1 may enable bacteria to sense extracellular NAD(P)^+^ released following lysis of nearby bacterial or eukaryotic cells, which may serve as a signal of danger (28, 47). Although this analysis is highly exploratory, our discovery of nontoxic bacterial ARC enzymes that synthesize appreciable quantities of cADPr lays the biochemical groundwork to better understand the role of this molecule in bacterial physiology.

## Experimental procedures

### Bacterial strains and growth conditions

*E*. *coli* strain XL1 Blue (Novagen) was used for plasmid maintenance, and strain BL21 (DE3) pLysS (Novagen) was used for recombinant protein expression. *E*. *coli* strains were grown in lysogeny broth (LB) medium (10 g/l tryptone, 5 g/l yeast extract, 10 g/l NaCl) at 37 °C shaking at 220 RPM, or on solid media containing 1.5% (w/v) agar. *E*. *coli* cultures were supplemented with 50 μg/ml kanamycin, 100 μg/ml ampicillin, 200 μg/ml trimethoprim, 15 μg/ml gentamicin, 0.1% l-rhamnose, 1 mM IPTG, as appropriate. A complete list of bacterial strains used in this study is available in Table S1.

### DNA manipulation and plasmid construction

All expression vectors were constructed using standard restriction enzyme–based cloning procedures (48). Primers were synthesized by Integrated DNA Technologies. Phusion polymerase, restriction enzymes, and T4 DNA ligase were obtained from New England Biolabs. Sanger sequencing was performed by The Center for Applied Genomics at the Hospital for Sick Children in Toronto, Canada. A complete list of plasmids used in this study is provided in Table S2.

### *E*. *coli* toxicity experiments and growth curves

Cultures of *E*. *coli* XL1 Blue harboring the indicated plasmids were grown overnight in 2 ml LB supplemented with appropriate antibiotics at 37 °C shaking at 220 RPM. Overnight cultures were normalized to an absorbance of 1.0 at 600 nm and were diluted in 10-fold series in a 96-well microtiter plate. About 6 μl of 10^-1^ to 10^-6^ dilutions were spotted on LB agar lacking or containing 0.1% (w/v) l-rhamnose or to induce toxin expression from pSCrhaB2-CV-derived vectors. Plates were incubated at 37 °C for 18 h and photographed.

Growth curves were conducted in liquid culture shaking at 220 RPM at 37 °C. Overnight cultures of *E*. *coli* XL1 Blue harboring the indicated plasmids were diluted to an absorbance of 0.05 at 600 nm in 200 μl LB supplemented with appropriate antibiotics in a 96-well microtiter plate. The plate was shaken at 37 °C in a BioTek Synergy H1 multimode reader (Agilent), and the absorbance at 600 nm was measured every 30 min. After 4 h of incubation, l-rhamnose and/or IPTG were added to a final concentration of 0.1% (w/v) or 1 mM respectively, and the plate was incubated for a further 12 h, with absorbance measurements every 30 min.

### Recombinant protein expression and purification

To protect *E*. *coli* from the toxic effects of Tac1 expression, Tac1 was coexpressed with its cognate immunity protein Tci1. Separation of the Tac1–Tci1 complex was achieved by denaturing with 8 M urea, as previously described (12). Briefly, *E*. *coli* BL21 (DE3) pLysS (Novagen) strains harboring expression vectors were grown in LB at 37 °C shaking at 220 RPM overnight with appropriate antibiotics. About 20 ml of starter culture was used to inoculate each 1 l LB, which was allowed to grow at 37 °C shaking at 220 RPM to an absorbance of 0.6 at 600 nm. IPTG was added at a final concentration of 1 mM to induce protein expression. The culture was subsequently incubated for 18 h at 18 °C shaking at 220 RPM before cells were harvested by centrifugation at 6000*g* for 20 min. Cells were resuspended in lysis buffer (300 mM NaCl, 50 mM Hepes, NaOH [pH 7.5]) and lysed by sonication. Lysates were clarified by centrifugation at 36,000 *g* for 45 min at 4 °C and loaded onto a 1 ml gravity flow nickel–nitrilotriacetic acid agarose (Qiagen) column pre-equilibrated with 10 ml wash buffer (300 mM NaCl, 50 mM Hepes [pH 7.5], 10 mM imidazole). The column was washed once with 20 ml wash buffer before nickel–nitrilotriacetic acid–bound Tac1–Tci1 complex was denatured with 20 ml 8 M urea. Denatured Tac1 was refolded by washing the column twice with 20 ml wash buffer before Tac1 was eluted by the addition of 4 ml elution buffer (300 mM NaCl, 50 mM Hepes [pH 7.5], 400 mM imidazole). Tac1 was further purified by gel filtration on a HiLoad 16/600 superdex 200 size-exclusion chromatography column (GE Healthcare) equilibrated with size-exclusion chromatography buffer (20 mM Hepes [pH 7.5], 150 mM NaCl). Protein purity was analyzed by SDS-PAGE stained with Coomassie Brilliant Blue R250. Protein concentration was measured using a NanoDrop instrument (ThermoFisher), and proteins were concentrated to 50 μM using a 10 kDa molecular weight cutoff centrifugal filter device (MilliporeSigma). Proteins were used immediately or snap frozen in liquid nitrogen and stored at −80 °C for later use. Tci1 was purified by the same method, omitting the denaturation step with 8 M urea.

### Crystallization and structure determination

Crystals were grown by the hanging drop vapor diffusion method at room temperature. To identify initial crystallization conditions, Tac1 crystallization trials were conducted using a 384-well sparse matrix screen (Microlytic). Trials were conducted in 48-well VDX plates (Hampton Research) by hand with 2.2 μl drops at a ratio of 1:1 protein to crystallization solution over a well containing 200 μl crystallization solution. Crystals appeared after 21 days of growth in 0.2 M NaCl, 0.1 M Bis–Tris HCl (pH 6.5), 25% (w/v) PEG 3350. Crystals were cryoprotected in crystallization solution supplemented with 40% (v/v) glycerol prior to vitrification.

Diffraction data were collected at National Synchrotron Light Source II in Brookhaven, NY, using the FMX 17ID-II beamline. Initial data processing and scaling were conducted using autoPROC (49) provided at the beamline. The structure was determined by molecular replacement and initially refined using MOLREP (50) and refmac5 (51) with the HKL3000 (52) software package, using a structural prediction generated by AlphaFold3 (40, 53). The initial model was built and refined manually using Coot (54) and automatically using Phenix.refine (55, 56). The final model was built in its entirety, except for the N-terminal His_6_ tag, to an *R*_work_/*R*_free_ of 0.170/0.197. The coordinates and structure factors (Protein Data Bank [PDB] ID: 9OUF) have been deposited to the PDB, Research Collaboratory for Structural Bioinformatics, Rutgers University, New Brunswick, NJ (www.rcsb.org and www.pdb.org).

### NAD(P)^+^ glycohydrolase assays

Rates of NAD(P)^+^ hydrolysis by Tac1 were determined using a fluorescent endpoint assay, as previously described (57). Triplicate 100-μl reactions in 20 mM Hepes (pH 7.5), 150 mM NaCl contained either 0.15 mM NAD^+^ or 0.15 mM NAD(P)^+^ and the following concentration of each enzyme: 0.1 nM Tac1 and Tac1^S544A^; 0.1 nM Tac1^W490A^, Tac1^516A^, Tac1^W529A^, Tac1^W540A^, Tac1^R564A^, Tac1^E573A^; or 100 nM Tac1–Tci1 complex. Each enzyme concentration was chosen to ensure <30% of the total substrate was depleted. Reactions were incubated at room temperature for 10 min and quenched by the addition of 50 μl 6 M NaOH. Unreacted NAD(P)^+^ remaining in each reaction was quantified by interpolation to a standard curve.

### Analysis of Tac1 and Nac1 reaction products by HPLC–MS

About 200 μl reactions of 1 mM NAD^+^ or NADP^+^ were incubated with 200 nM Tac1 or the indicated Tac1 variants in 10 mM ammonium acetate for 10 min at 25 °C. Reactions were stopped by the addition of 200 μl methanol and centrifuged at 21,000*g* to pellet precipitated protein. Reaction products were analyzed in an LC–MS system including an Agilent 1200 series HPLC system coupled with a Bruker MicroTOF II mass spectrometer. For each run, 5 μl of sample was injected onto a normal phase hydrophilic interaction liquid chromatography column at a flow rate of 0.5 ml/min, using a mobile phase consisting of 0.1% (w/v) formic acid (A) and acetonitrile (B). Reaction products were eluted using a linear gradient that began at 95% (B) and ended at 12 min with 5% (B). The mobile phase was held for 3 min at 5% (B), before the column was re-equilibrated with 95% (B) for 5 min. NAD^+^, NAD(P)^+^, ADP-ribose, and ADP-ribose 2′-phosphate were ionized by electrospray ion source, using N_2_ for nebulization (pressure of 2.0 Bar) and drying (flow of 6 l/min, temperature of 200 °C). Capillary voltage was 4500 V, end plate offset −500 V, hexapole radio frequency of 450.0 Vpp, energy transfer time of 49.0 μs, and prepulse storage of 10.0 μs. MS data were collected in positive electrospray ionization mode. Data were acquired in centroid mode spanning an *m/z* range of 200 to 1000. The resulting spectra were analyzed using Agilent Mass Hunter qualitative analysis software.

### Bioinformatic identification of bacterial ARC enzymes

To identify the gene neighborhoods in which bacterial ARC proteins are encoded, we downloaded all bacterial proteins in the RefSeq protein database with the annotation CL08346 (ADP-ribosyl cyclase) and analyzed the genes flanking these proteins using FlaGs (58). In addition, we queried the sequence of the toxin domain of Tac1 (residues 458–602) against the Genome Taxonomy Database using AnnoView (35).

## Data availability

The X-ray crystallographic structure of Tac1 was deposited in the PDB with the ID 9OUF.

## Supporting information

This article contains supporting information (59, 60, 61).

## Conflict of interest

The authors declare that they have no conflicts of interest with the contents of this article.
