# Supplementary material for: Antibacterial ADP-ribosyl cyclase toxins inhibit bacterial growth by rapidly depleting NAD(P)+
Source: J Biol Chem. 2025 Jul 16;301(8):110491. doi: 10.1016/j.jbc.2025.110491 (PMC12356396; doi:10.1016/j.jbc.2025.110491)
Supplement: Supplementary Material [file mmc1.pdf]

Supplementary Material

Antibacterial ADP-ribosyl cyclase toxins inhibit bacterial growth by rapidly depleting NAD(P)$^+$

Jake Colautti[1,2], Youngchang Kim, and John C. Whitney[1,2,3]*

[1]Michael DeGroote Institute for Infectious Disease Research, McMaster University, Hamilton, ON, L8S 4K1, Canada
[2]Department of Biochemistry and Biomedical Sciences, McMaster University, Hamilton, ON, L8S 4K1, Canada
[3]David Braley Center for Antibiotic Discovery, McMaster University, Hamilton, ON, L8S 4K1, Canada

Supplementary Figures S1-S4
Supplementary Tables S1-S2
Supplementary References

Keywords: Bacterial toxin, cyclic ADP-ribose, X-ray crystallography, enzyme kinetics, toxin secretion

*To whom correspondence should be addressed: John C Whitney
Email – jwhitney@mcmaster.ca
Telephone – (+1) 905-525-9140

Figure S1: AlphaFold3 predicts that Tci1 interacts with the catalytic cleft of Tac1. AlphaFold3-predicted model of Tac1 (green) in complex with its cognate immunity protein Tci1 (pink). The predicted aligned error (PAE) plot is shown at the top right.

Figure S2: HPLC/MS identification of the Tac1 cADPr catalytic intermediate. A) UV absorbance HPLC chromatograms of the reaction products of NAD$^+$ incubated with the indicated Tac1 variants. NAD$^+$ and cADPr standards are shown to determine the retention time of each species. B) Zoomed view of the UV chromatograms in (A) to highlight the presence of the cADPr peak in the reaction catalyzed by Tac1^{E516A}. C) Mass spectra of the products of the reaction catalyzed by Tac1^{W540A} and Tac1^{E516A} at a retention time of 6.748 mins, corresponding to the rention time of cADPr. The *m/z* of each major species is indicated.

Figure S3: T6SS-associated Tac1 homologs are predicted to bind to distinct immunity proteins. AlphaFold3-predicted model of a Tac1 homolog associated with a T6SS in *Duganella vulcanian* (green and blue) in complex with the putative immunity protein encoded downstream (purple). The predicted aligned error (PAE) plot is shown at the top right.

Figure S4: *A. oryzae* Nac1 contains a predicted N-terminal Sec signal peptide. SignalP server output for the full-length sequence of *A. oryzae* Nac1 (Teufel *et al*, 2022).

Table S1: Strains used in this study

Organism	Genotype	Description	Reference
E. coli XL-1 Blue	*recA1 endA1 gyrA96 thi-1 hsdR17 supE44 relA1 lac* [F′ *proAB lacI*^{q} Z*ΔM15* Tn*10* (Tet^R)]	Cloning strain	Novagen
E. coli BL21 (DE3) pLysS	F- ompT gal dcm lon hsdSB(rB$^-$ mB$^-$) λ(DE3) pLysS(cm^R)	Protein expression strain	Novagen

Table S2: Plasmids used in this study

Plasmid	Relevant features	Reference
pSCrhaB2-CV	Expression vector with P*rhaB*, C-terminal VSV-G tag, TmpR	(Cardona & Valvano, 2005)
pSCrhaB2-CV:: HMPREF8577_0804_458-CT	Tac1 expression vector	This study
pSCrhaB2-CV:: HMPREF8577_0804_458-CT_W490A	Tac1^{W540A} expression vector	This study
pSCrhaB2-CV:: HMPREF8577_0804_458-CT_E516A	Tac1^{E516A} expression vector	This study
pSCrhaB2-CV:: HMPREF8577_0804_458-CT_W529A	Tac1^{W529A} expression vector	This study

pSCrhaB2-CV:: HMPREF8577_0804_458-CT_W540A	Tac1^{W540A} expression vector	This study
pSCrhaB2-CV:: HMPREF8577_0804_458-CT_S544A	Tac1^{S544A} expression vector	This study
pSCrhaB2-CV:: HMPREF8577_0804_458-CT_R564A	Tac1^{S564A} expression vector	This study
pSCrhaB2-CV:: HMPREF8577_0804_458-CT_E573A	Tac1^{W573A} expression vector	This study
pETDuet-1	Co-expression vector with *lacI*, T7 promoter, N-terminal His$_6$ tag in MCS-1, AmpR	Novagen
pETDuet-1:: HMPREF8577_0804_458-CT:: HMPREF8577_0803	Tac1/Tci1 co-expression vector	This study
pETDuet-1:: HMPREF8577_0804_458-CT_W490A:: HMPREF8577_0803	Tac1^{W490A}/Tci1 co-expression vector	This study
pETDuet-1:: HMPREF8577_0804_458-CT_E516A:: HMPREF8577_0803	Tac1^{E516A}/Tci1 co-expression vector	This study
pETDuet-1:: HMPREF8577_0804_458-CT_E529A:: HMPREF8577_0803	Tac1^{E529A}/Tci1 co-expression vector	This study
pETDuet-1:: HMPREF8577_0804_458-CT_W540A:: HMPREF8577_0803	Tac1^{W540A}/Tci1 co-expression vector	This study
pETDuet-1:: HMPREF8577_0804_458-CT_S544A:: HMPREF8577_0803	Tac1^{S544A}/Tci1 co-expression vector	This study
pETDuet-1:: HMPREF8577_0804_458-CT_R564A:: HMPREF8577_0803	Tac1^{R564A}/Tci1 co-expression vector	This study
pETDuet-1:: HMPREF8577_0804_458-CT_E573A:: HMPREF8577_0803	Tac1^{E573A}/Tci1 co-expression vector	This study
pET28b	Expression vector with *lacI*, T7 promoter, N-terminal His$_6$ tag, KanR	Novagen
pET28b::HMPREF8577_0803	Tci1 expression vector	This study
pPSV39-CV	Expression vector with *lacI*, *lacUV5* promoter, GmR	(Silverman *et al*, 2013)
pPSV39-CV::HMPREF8577_0803	Tci1 expression vector	This study

Supplementary References

Cardona ST, Valvano MA (2005) An expression vector containing a rhamnose-inducible promoter provides tightly regulated gene expression in Burkholderia cenocepacia. *Plasmid* 54: 219-228

Silverman JM, Agnello DM, Zheng H, Andrews BT, Li M, Catalano CE, Gonen T, Mougous JD (2013) Haemolysin coregulated protein is an exported receptor and chaperone of type VI secretion substrates. *Mol Cell* 51: 584-593

Teufel F, Almagro Armenteros JJ, Johansen AR, Gislason MH, Pihl SI, Tsirigos KD, Winther O, Brunak S, von Heijne G, Nielsen H (2022) SignalP 6.0 predicts all five types of signal peptides using protein language models. *Nat Biotechnol* 40: 1023-1025
