# Supplementary figures and images for: Antibacterial ADP-ribosyl cyclase toxins inhibit bacterial growth by rapidly depleting NAD(P)+
Source: J Biol Chem. 2025 Jul 16;301(8):110491. doi: 10.1016/j.jbc.2025.110491 (PMC12356396; doi:10.1016/j.jbc.2025.110491)

Figure S1

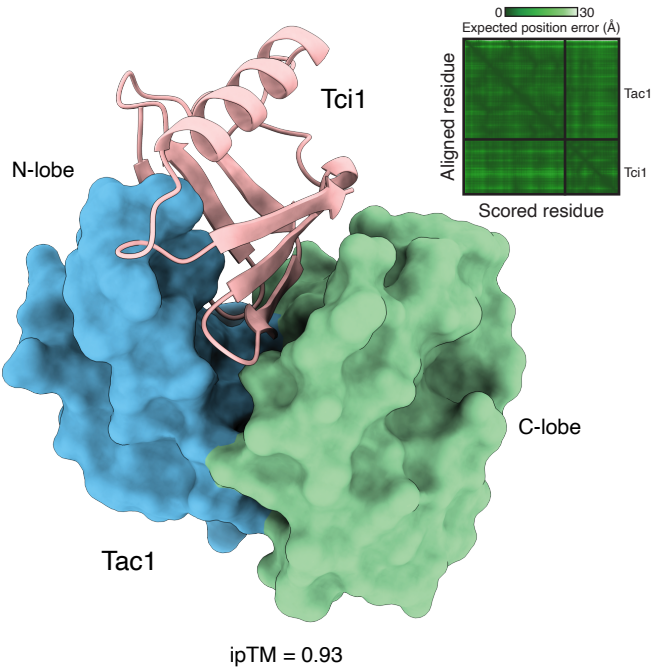

Supplement: Figure S1 [file mmc2.pdf]

Figure S2

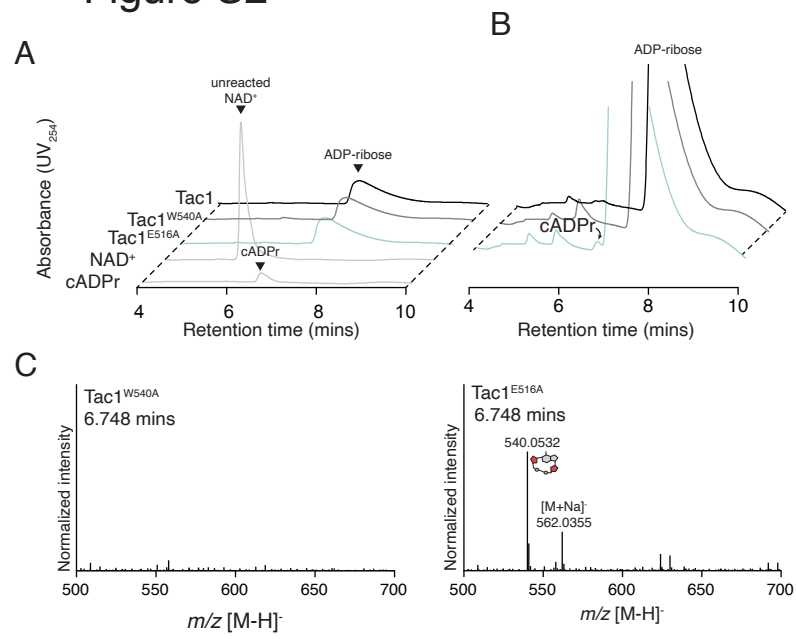

Supplement: Figure S2 [file mmc3.pdf]

Figure S3

A

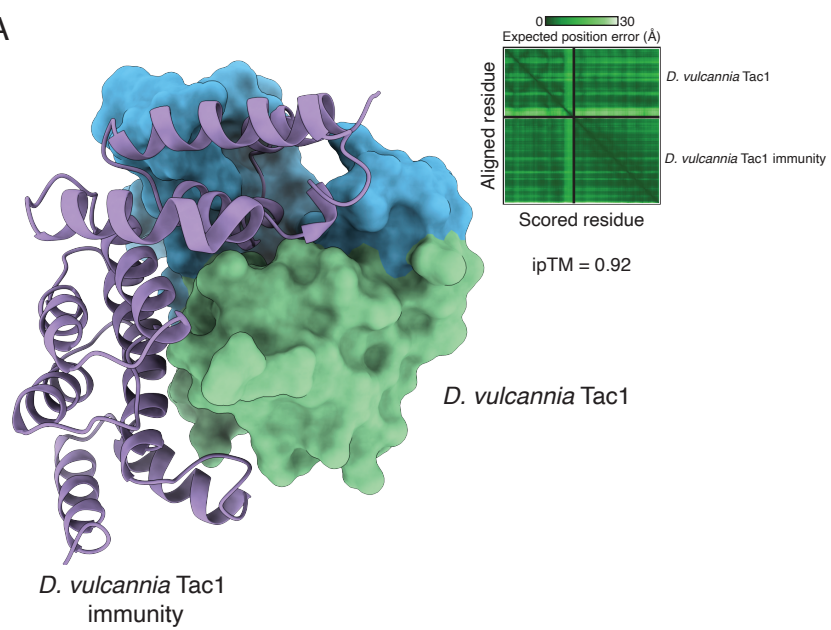

Supplement: Figure S3 [file mmc4.pdf]

Figure S4

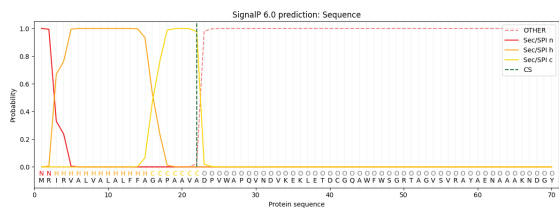

Supplement: Figure S4 [file mmc5.pdf]
